# Supplementary material for: The High-Throughput In Vitro CometChip Assay for the Analysis of Metal Oxide Nanomaterial Induced DNA Damage
Source: Nanomaterials (Basel). 2022 May 27;12(11):1844. doi: 10.3390/nano12111844 (PMC9181865; doi:10.3390/nano12111844)
Supplement: Supplementary file 1 [file nanomaterials-12-01844-s001.zip › nanomaterials-1683268-Figures and Tables.pdf]

nanomaterials

Article

The High-Throughput In Vitro CometChip Assay for the Analysis of Metal Oxide Nanomaterial Induced DNA Damage

Andrey Boyadzhiev, Silvia Aidee Solorio-Rodriguez, Dongmei Wu, Mary-Luyza Avramescu, Pat Rasmussen and Sabina Halappanavar *

Environmental Health Science and Research Bureau, Health Canada, Ottawa, ON K1A 0K9, Canada; andrey.boyadzhiev@hc-sc.gc.ca (A.B.); silvia.solorio-rodriguez@hc-sc.gc.ca (S.A.S.-R.); dongmei.wu@hc-sc.gc.ca (D.W.); mary-luyza.avramescu@hc-sc.gc.ca (M.-L.A.); pat.rasmussen@hc-sc.gc.ca (P.R.)

* Correspondence: sabina.halappanavar@hc-sc.gc.ca

Table S1. Sonication parameters for the metal oxides examined in this study. For all materials, the volume of the stock suspensions was ~8 mL. The sonicator was operated at 80 % cycles in all instances (8 seconds on with 2 seconds off). DSE: delivered sonication energy.

Metal Oxide	Concentration (mg/mL)	DSE (J/mL)	% Amplitude	Total Sonication Time (s)
ZnO	5	655	40	135
CuO	1	109.5	10	114
TiO_2	5	1013	55	144

Table S2. Dose interconversions for pristine MONM, MP, and metal chloride concentrations used for *in vitro* exposure.

Metal Oxide	Mass Dose (μg/mL)	Well-Surface Area Dose (μg/cm^2)	Metal Chloride Dose (μg/mL)
ZnO	5	1.56	8.5[a]
	10	3.13	16.5[a]
	20	6.25	33.5[a]
	30	9.38	50[a]
	40	12.50	67[a]
CuO	1	0.31	1
	5	1.56	7
	10	3.13	22[a]
	25	7.81	54[a]
	50	15.63	108[a]
TiO_2	6.25	1.95	N/A
	12.5	3.91	N/A
	25	7.81	N/A
	50	15.63	N/A
	100	31.25	N/A

[a]: Doses contain metal molar equivalents of the respective MONM dose.

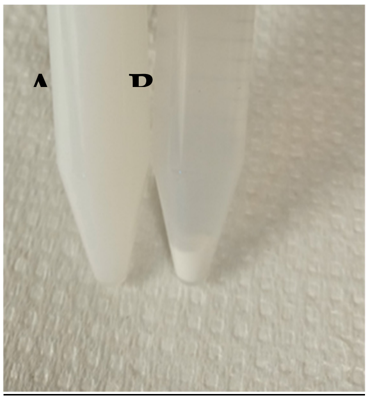

Figure S1. MK-TiO_2-A050 NPs suspension at 1 mg/mL in DMEM supplemented with 2% FBS A) with or B) without 0.4 mg/mL BSA.

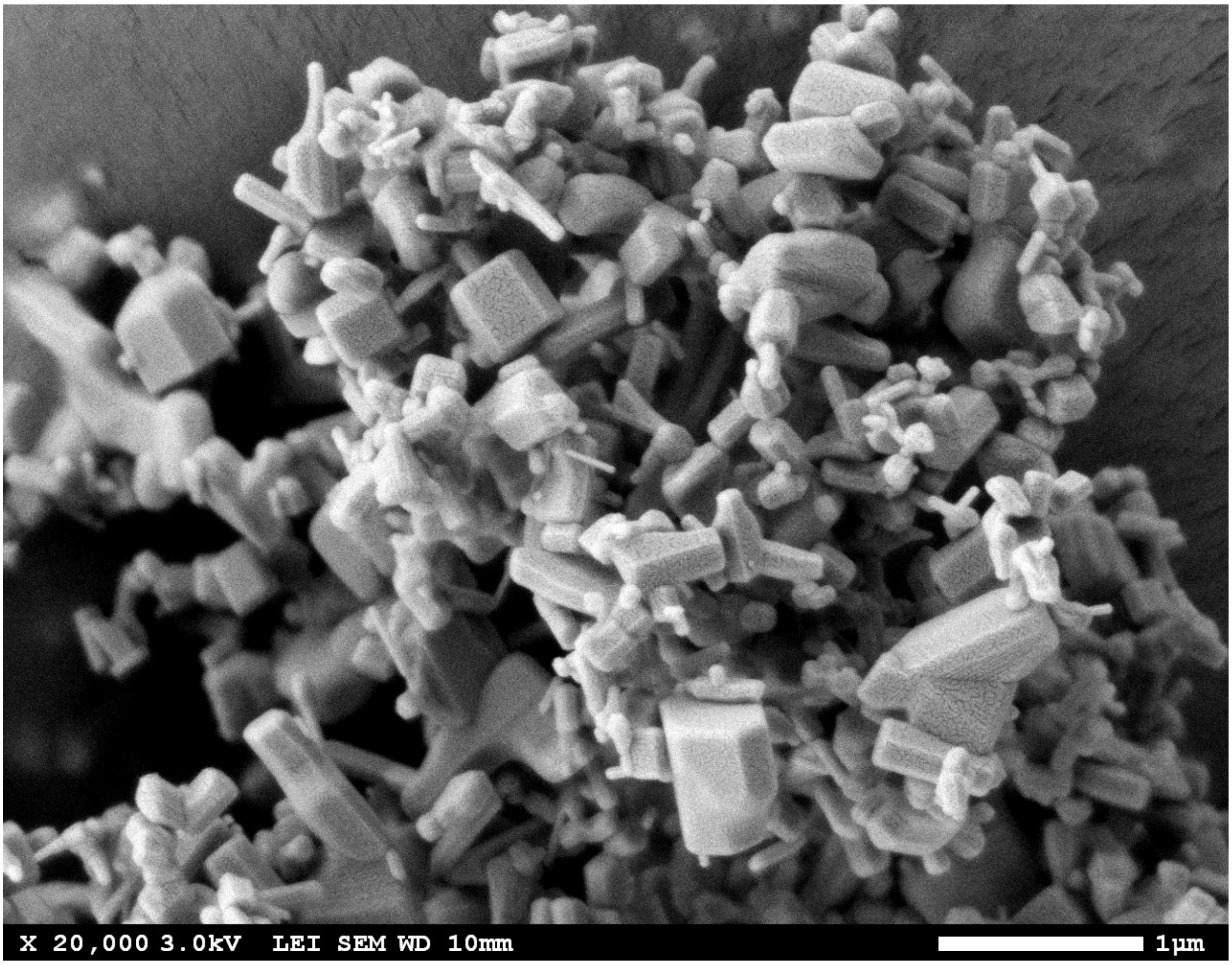

Figure S2. Representative scanning electron micrograph showing the morphology of the ZnO MPs used in this study.

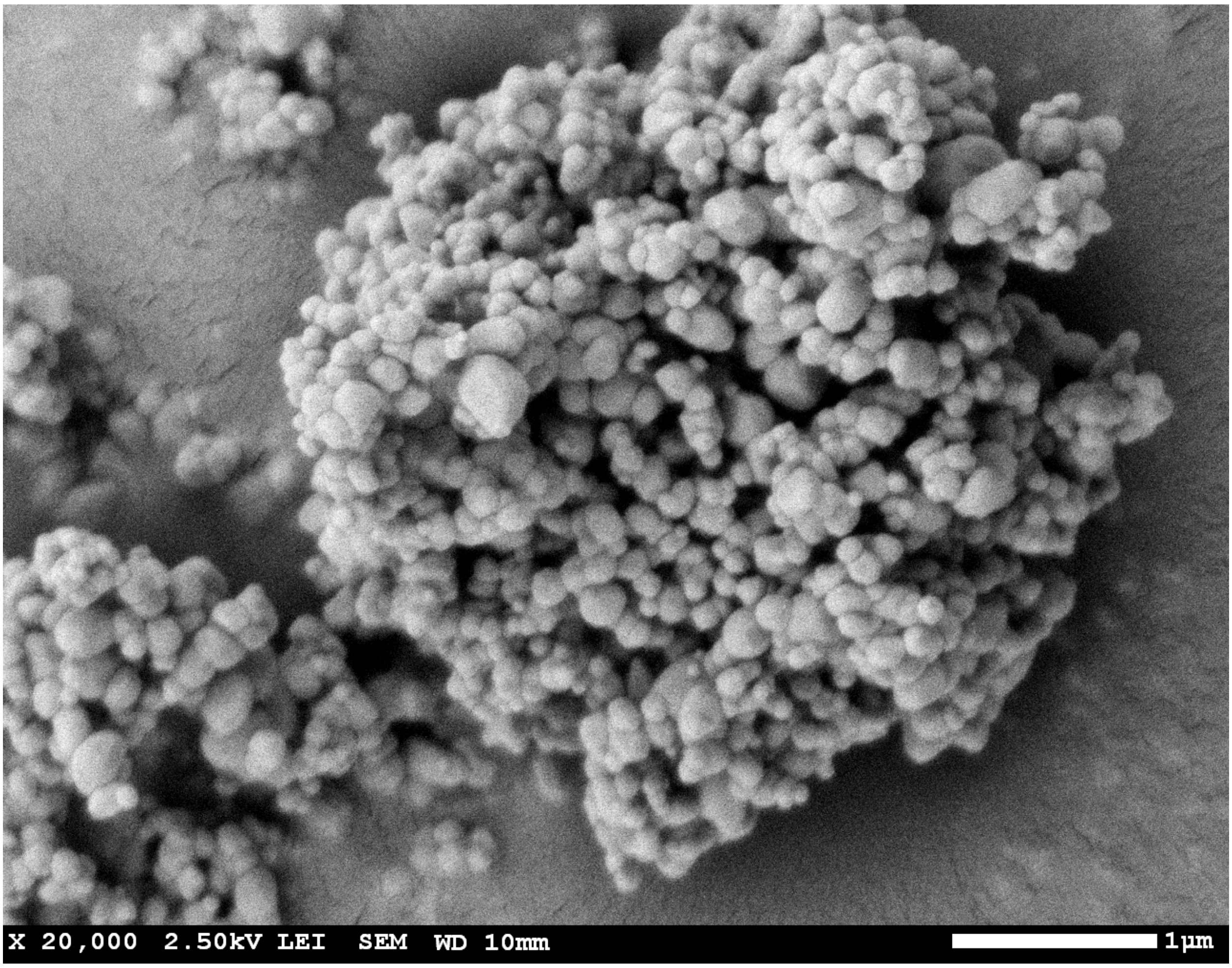

Figure S3. Representative scanning electron micrograph showing the morphology of the TiO_2 MPs used in this study.

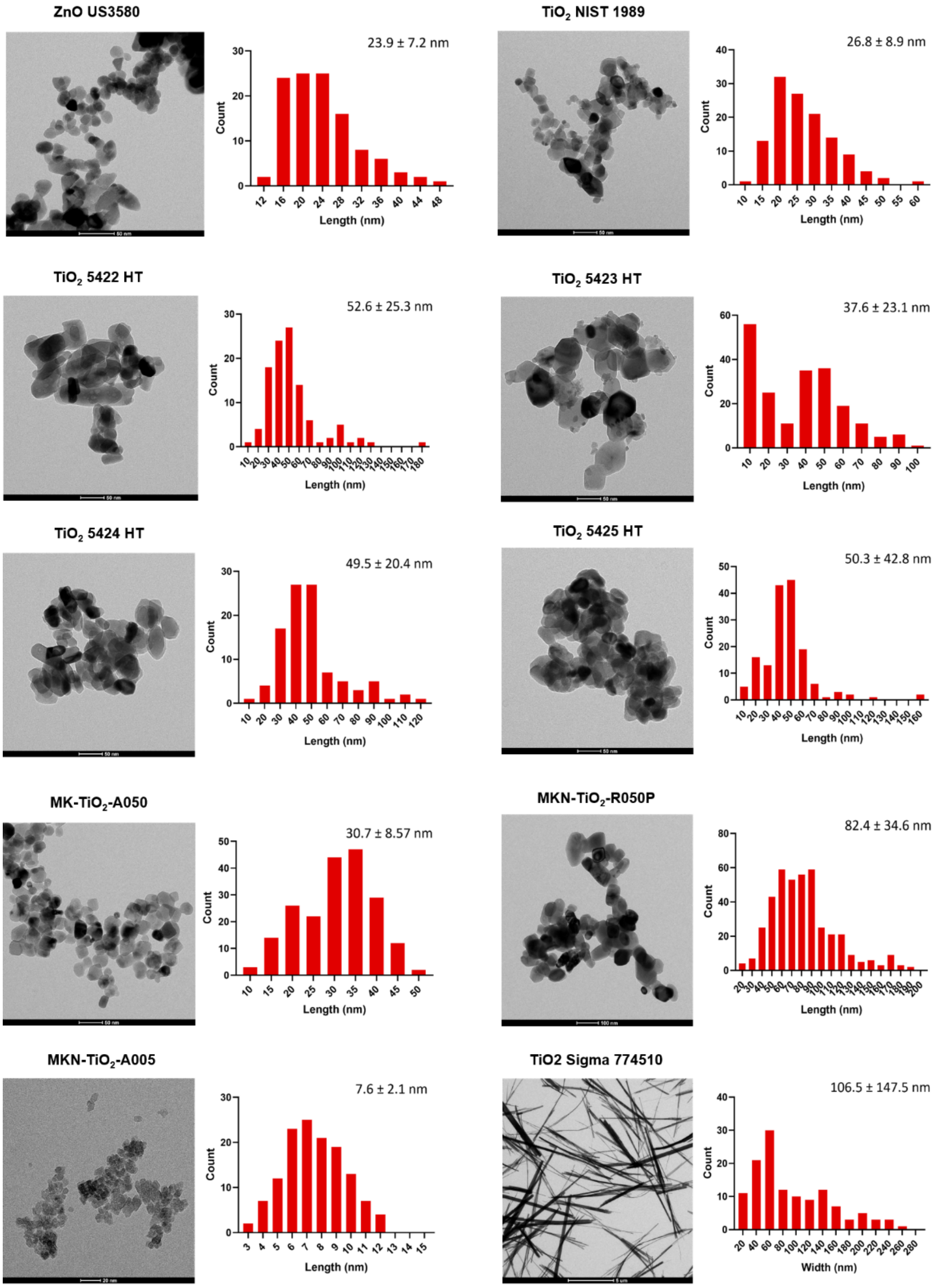

Figure S4. Transmission electron microscopy and primary particle size distribution of different TiO_2 NPs and one type of TiO_2 NWs used in this study (n = 100–250). Distributions of primary particle length and width were determined using ImageJ. Data represent mean particle length, +/- standard deviation. For non-spherical nanoparticles (MKN-TiO2-R050P), the mean width is also shown. For TiO_2 nanowires (Sigma 774510), the width is shown as the length was not possible to measure due to their tangled nature.

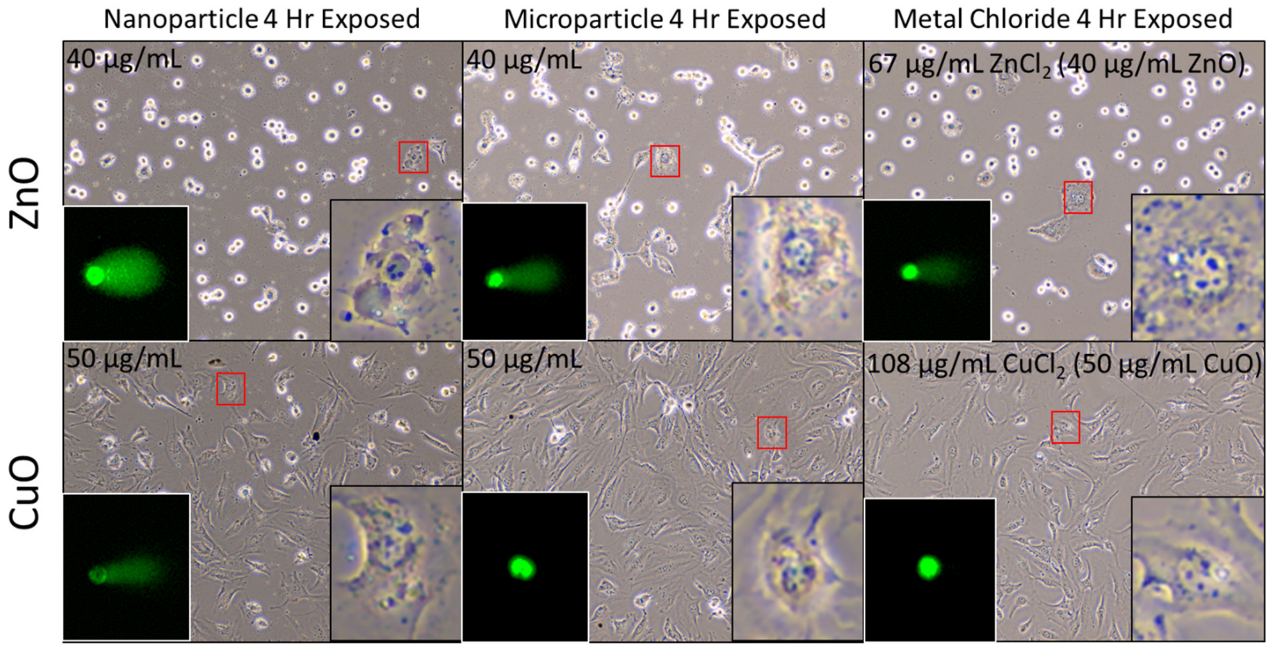

Figure S5. Phase contrast images (20X) and representative fluorescent comet micrographs of FE1 cells after 4 Hr exposure to ZnO & CuO NPs, MPs, and metal chloride equivalents. Insets on the bottom right of the micrographs represent an enlargement of the areas highlighted in red. Comet micrographs had brightness and contrast adjusted to aid in visualization.

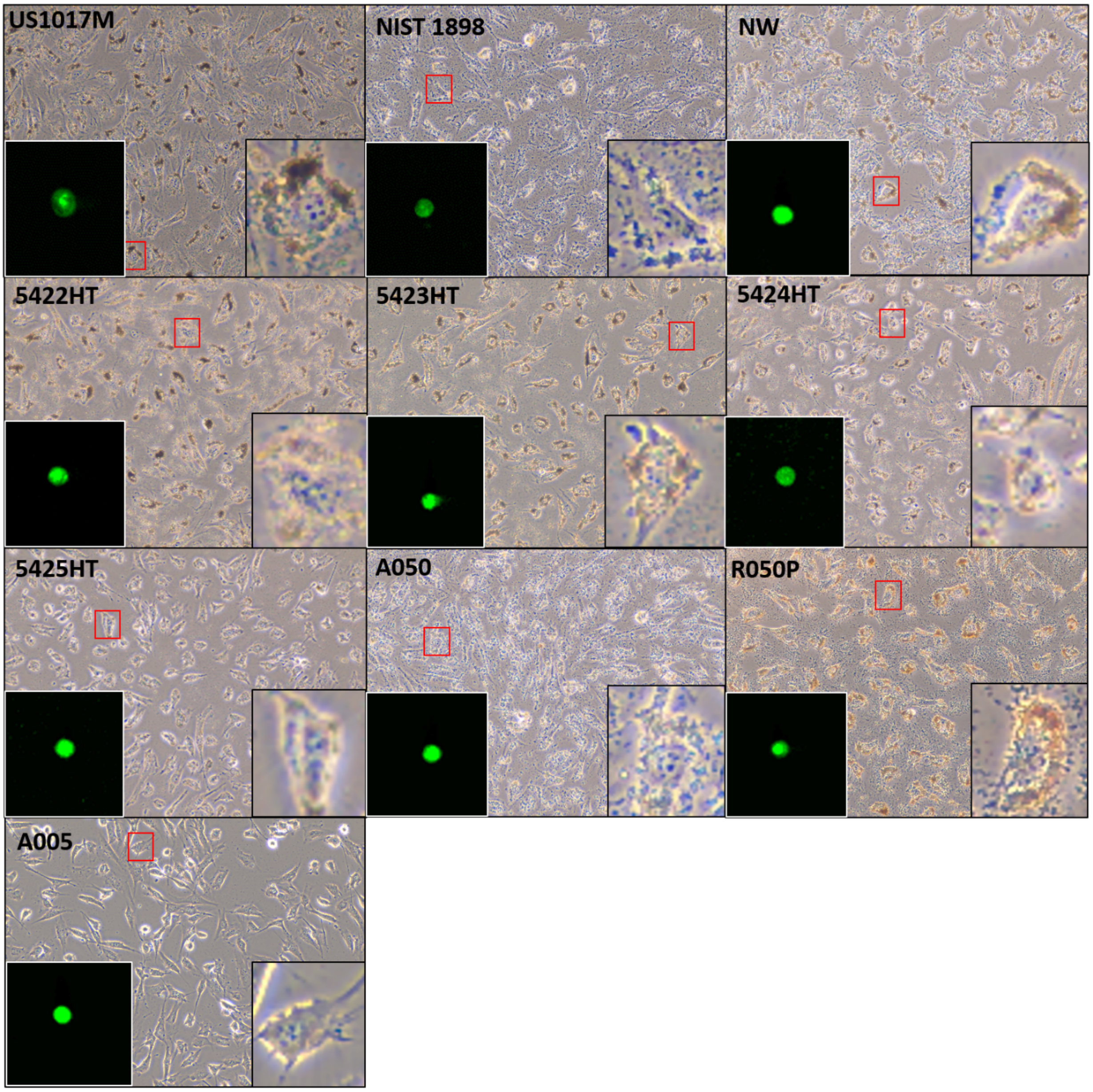

Figure S6. Phase contrast images (20X) and representative fluorescent comet micrographs of FE1 cells after 4 Hr exposure to 100 µg/mL TiO_2 nanomaterials and microparticles. NW: nanowires. Insets on the bottom right of the micrographs represent an enlargement of the areas highlighted in red. Comet micrographs had brightness and contrast adjusted to aid in visualization.
